# Modulation of neural activity and gene expression by arecoline

**DOI:** 10.3389/fnint.2025.1545260

**Published:** 2025-04-09

**Authors:** Xiaonan Li, Jie Gao, Xiaomin Liu, Jianfeng Guo, Yifan Liu, Peicai Cui, Dawei Yan, Ting Fei, Ming Chen, Yihan Gao

**Affiliations:** ^1^Shanghai New Tobacco Products Research Institute Co., Ltd., Shanghai, China; ^2^Fudan University, Shanghai, China

**Keywords:** arecoline, conditioned place preference, 3D behavioral analyses, transcriptomic analyses, hippocampus

## Abstract

Arecoline, a biologically active alkaloid extracted from the areca nut, serves as the primary psychoactive ingredient in betel quid, one of the most widely consumed psychoactive substances worldwide. Despite its extensive use, the central nervous system (CNS) effects of arecoline remain inadequately understood. This study aims to investigate the central actions of arecoline through a comprehensive, multi-dimensional approach that integrates behavioral assays, neuroimaging techniques, calcium signaling analysis, and transcriptomic profiling. Our findings demonstrate dose-dependent addictive properties of arecoline, alongside distinct behavioral alterations that highlight its potential for addiction. Neuroimaging and calcium signaling data revealed region-specific alterations in neural activity, particularly in areas associated with learning, memory, and reward processing. Furthermore, transcriptomic analysis identified significant changes in gene expression, particularly in pathways related to synaptic plasticity, calcium signaling, and metal ion transport. These results provide valuable insights into the addictive potential of arecoline and its underlying neurobiological mechanisms, offering crucial information for understanding its broader impact on CNS function. The study’s findings hold significant implications for informing public health strategies aimed at addressing arecoline misuse and its potential role in addiction-related disorders.

## Introduction

Arecoline is a naturally occurring alkaloid found in the seeds of the areca palm, commonly known as betel nuts (Athukorala et al., 2021). It serves as the primary psychoactive component in betel quid, a traditional substance widely consumed across Asia and the Pacific Islands (Kong et al., 2020). Habitual betel quid chewing is the fourth most prevalent psychoactive behavior globally, following the use of alcohol, caffeine, and nicotine (Papke et al., 2015). Despite its broad cultural and social significance, the central mechanisms underlying arecoline’s actions remain incompletely understood, particularly concerning its potential impact on addiction-related behaviors.

Pharmacologically, arecoline exhibits a complex profile of activity. As a partial agonist of muscarinic acetylcholine receptors (mAChRs) (Johnson et al., 2022; Das and Giri, 2020), it modulates cholinergic signaling, influencing both peripheral and central nervous system (CNS) functions (Picciotto et al., 2012). Additionally, arecoline interacts with nicotinic acetylcholine receptors (nAChRs) (Yohn et al., 2022; Horenstein et al., 2019), particularly subtypes associated with addiction, such as α4β2 and α6β3-containing nAChRs (Liu and Li, 2018). Notably, it also acts as a silent agonist at α7 nAChRs, with its activity potentiated by positive allosteric modulators (Gill et al., 2011). These diverse receptor interactions are believed to underlie the compound’s ability to affect mood (Feduccia et al., 2012; Mineur et al., 2016), cognition (Echeverria et al., 2023), and neural plasticity (McKay et al., 2007).

Compared to nicotine, a prototypical nAChR agonist with well-documented addictive potential, arecoline’s addiction liability appears lower (Papke et al., 2015). The study shown arecoline produced the highest CPP response in adolescent or adult mice, respectively (Pi et al., 2023). Arecoline’s psychoactive effects may not heavily rely on modulating synaptic plasticity in the NAc (Huang et al., 2025). However, its regular use among betel quid chewers, often accompanied by psychological dependence, suggests that it may contribute to addictive behaviors through distinct or overlapping mechanisms. Moreover, emerging evidence indicates that arecoline may exert anti-inflammatory effects and influence synaptic plasticity (Chen et al., 2024; Tong et al., 2024), potentially linking its neurobiological actions to both therapeutic and adverse outcomes.

Understanding the central actions of arecoline is particularly important in the context of rising interest in novel alkaloid-based substances, including its potential incorporation into new-generation tobacco products (Yach and Scherer, 2023). Research into arecoline’s neurobiological effects provides a unique opportunity to uncover how alkaloids modulate neural circuits, impact behavior, and interact with molecular pathways in the CNS.

This study aims to investigate the central actions of arecoline through behavioral paradigms, neural circuit analyses, and molecular approaches. Using a combination of techniques such as conditioned place preference (CPP), advanced behavioral monitoring, brain imaging, calcium signaling studies, and transcriptomic profiling, this research seeks to elucidate the addictive potential, neural modulation, and molecular mechanisms underlying arecoline’s effects. The findings will contribute to a deeper understanding of arecoline’s central actions, offering insights into its role within the broader context of alkaloid-induced neuropharmacology.

## Materials and methods

### Experimental animals

Adult male mice were C57BL/6 mice (8 to 10 weeks of age) weighing 22–28 g (Shanghai Jihui Experimental Animal Breeding Co., Ltd.), which were housed for at least 1 week in advance in an animal room with alternating cycles of 12 h of light and 12 h of darkness (lights on at 7:00 am and off at 7:00 pm), at a temperature of 22–26°C and 40–60% humidity. All mice had free access to food and water. All experimental procedures were approved by the Animal Ethics and Use Committee of the Fudan University (approval number: 20241107-001) and were performed in accordance with NIH guidelines. Mice were randomly divided into cages and labeled according to experimental groups. All behavioral tests were carried out in the same room environment, and ambient conditions such as light intensity, temperature, and humidity were kept consistent.

### Conditioned place preference

The conditioned place preference (CPP) model was utilized to assess the rewarding properties of the alkaloids (Cata-Preta et al., 2018). The experimental procedure began with a preconditioning phase where animals were acclimated to the laboratory environment and allowed to explore the CPP apparatus freely for 15–20 min to reduce novelty-induced stress and identify their natural compartment preferences. This phase served to establish a baseline for comparison during subsequent testing. In the conditioning phase, animals received intraperitoneal injections of alkaloid solutions or saline according to their group assignment. During morning sessions, arecoline-treated animals were confined to their non-preferred compartments for 60 min to associate the environment with drug-induced euphoria. In the evenings, saline injections were paired with the preferred compartments to attenuate natural bias. This process was repeated for 5 days. In the postconditioning phase, animals were allowed to explore the apparatus freely without any treatment. Time spent in each compartment was recorded, and the CPP score, defined as the difference in time between compartments, was calculated. Higher CPP scores indicated successful addiction modeling.

### The 3D-motion capture system

The 3D-motion capture system was used to capture the movements of mice during the spontaneous behavioral test (Huang et al., 2021). The open-field box is made up of a transparent acrylic wall that stands 30 cm tall and a white plastic square floor with sides measuring 40 cm in length. Although a small cuboid (15 cm in length, 18 cm in width, and 15 cm in height) was present in one corner of the open field box, an acrylic transparent partition was placed at the junction of the two boxes to prevent the mouse from accessing the small cuboid free. The open field arena was positioned at the center of a movable stainless-steel support framework measuring 130 × 130 × 90 cm^3^. The framework had four Intel RealSense D435 cameras mounted orthogonally on its four supporting pillars, and a 56-inch screen was placed horizontally but face down on the top of the shelf to provide uniform and stable white background light. Animals’ behavioral data were extracted from 16 key body parts, including the nose, left ear, right ear, neck, left front limb, right front limb, left hind limb, right hind limb, left front claw, right front claw, left hind claw, right hind claw, back, root tail, middle tail, and tip tail. These key points were used to reconstruct 3D skeletons. The detailed methods, including camera calibration, and animals’ behavioral image acquisition, of this 3D multi-view motion-capture system setup (BA-DC01, Shenzhen Bayone BioTech Co., Ltd., Shenzhen), were described in our previous work.

### Immunostaining

Ninety minutes after the final CPP test, animals were perfused with 4% paraformaldehyde, and their brains were sectioned into 40-μm slices. Brain slices were blocked and incubated with primary antibodies targeting the c-Fos protein (Bullitt, 1990), followed by secondary and tertiary antibody incubations. Stained slices were visualized using fluorescence microscopy, and c-Fos-positive cells in key brain regions were quantified to evaluate neuronal activation across groups.

### Stereotaxic surgery

The surgical procedure was as follows: the mice were anesthetized with isoflurane; the surgery was performed under a continuous gas mixture of oxygen and isoflurane. The mouse’s head was adjusted to a horizontal position by Bregma and Lambda points on a stereotaxic instrument. Small holes were drilled above the mPFC brain region (AP: 1.8, ML: 0.5, DV: 1.5), and bleeding was promptly stopped if it occurred. The virus was injected as a dopamine neurotransmitter probe: rAAV-hSyn-GCaMP7-WPRE-pA, with an injection volume of 300 nL and an injection rate of 30–50 nL/min, and the needle was stopped for 10 min after the injection to allow complete virus diffusion. The optical fiber was buried above the NAc brain region of the mice with a specific optical fiber holder, and the optical fiber was fixed with dental cement. After the dental cement was completely dried, the mice were removed from the operating table and when the mice were awakened, they were marked and put back into the feeding cage.

### Fiber photometry recording

After 3 weeks of virus expression, the fiber optic patch cord of the fiber optic recording system was fixedly connected to the ceramic insert to test the fluorescent signal and animals that responded well were prepared for test. GCaMP7 signals were observed in mice before and after injection of caffeine (Li et al., 2019). The value of Δ*F*/*F* = (*F* − *F*_0_)/*F*_0_ was used to characterize the change in fluorescence intensity around the event as a response to the change in neuron activity under caffeine. The recorded data were exported as mat files for further analysis in Matlab software, first preprocessed by steps such as (1) baseline calibration (2) down sample (3) smooth. The signal change was calculated following the following formula: Δ*F*/*F* = (*F* − *F*_0_)/(*F*_0_ − *F*_offset_). The significance of the parameters in this formula is as follows: *F* represents the current signal value, *F*_0_ represents the mean signal value 15 min before drug administration; *F*_offset_ represents the background noise value of the instrument; Δ*F*/*F* represents the relative signal change value, expressed as a percentage, and the percentage signal change before and after stimulation was plotted for a single mouse using the Matlab software plot function.

### Transcriptomic analysis

After the CPP tests, brain tissues from specific regions were rapidly dissected and flash-frozen. RNA was extracted and processed to construct cDNA libraries, which were sequenced using high-throughput technology (Crist et al., 2021). Differentially expressed genes were identified, and pathway enrichment analyses were performed to map the molecular pathways and networks involved in alkaloid-induced addiction.

### Analysis

Numerical data were expressed as mean ± SEM. Off-line data analysis was performed using software GraphPad Prism 6 (GraphPad Software, United States). Statistical significance was determined by ANOVA followed by Bonferroni post-tests for multiple comparisons among more than two groups. *n* refers to the number of mice. Every group of mice in each experiment was from at least five animals. For all results, *p* < 0.05 was accepted as being statistically significant.

## Results

### Behavioral analysis of arecoline-induced conditioned place preference and 3D behavioral analysis

**Figure 1:**
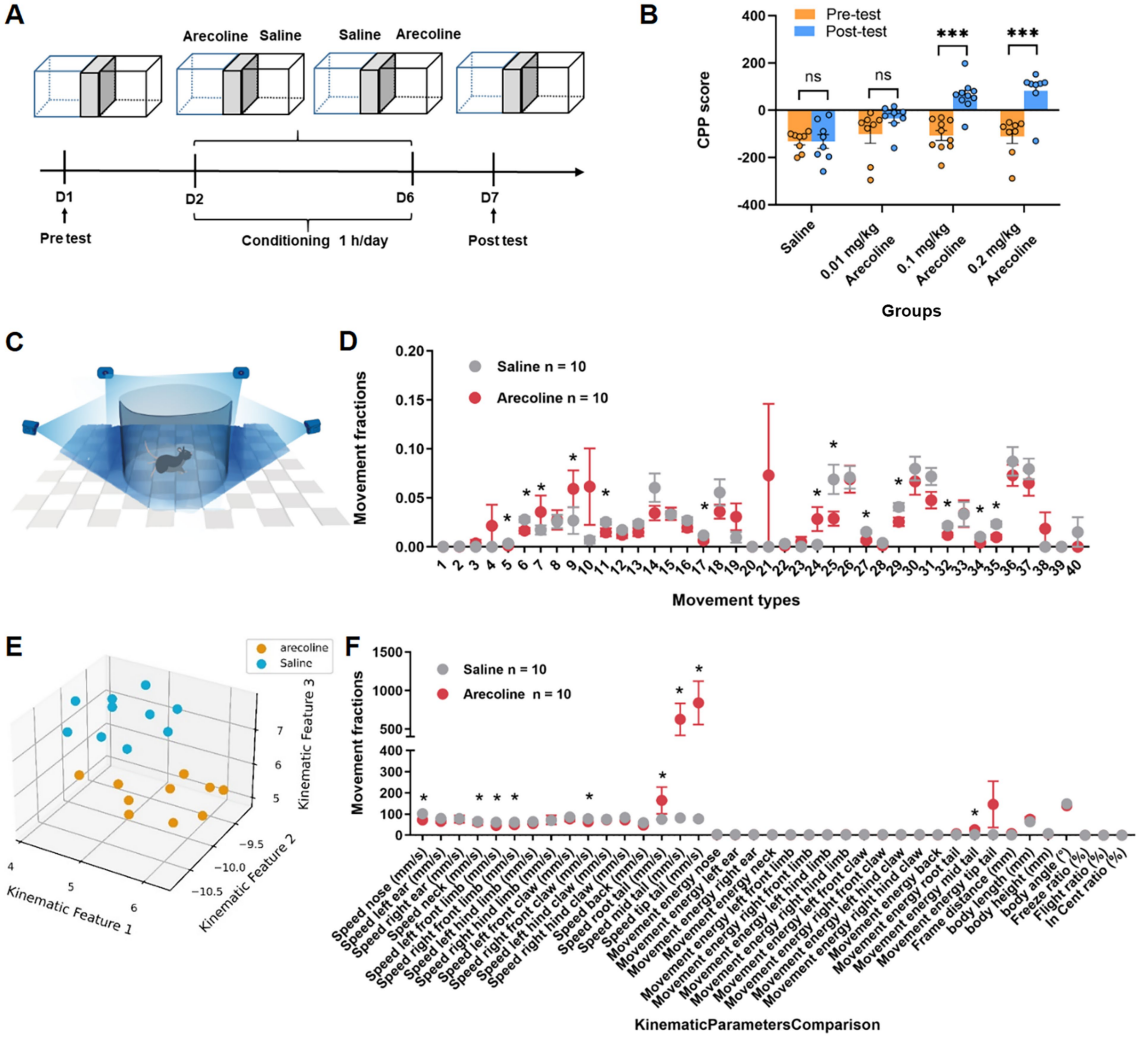
Behavioral effects of arecoline in adult male mice. **(A)** Schematic of the experimental setup. **(B)** The average CPP score in three dose of arecoline groups (two-way ANOVA, Sidak’s multiple comparisons test, the low dose, *p* > 0.05, the medium dose, *p* < 0.0001, the high dose, *p* < 0.0001, *n* = 8 in 0.01 mg/kg dose group, *n* = 10 in 0.1 mg/kg dose group, *n* = 8 in 0.2 mg/kg dose group). **(C)** Schematic diagram of recording animal behavior with four synchronized cameras. **(D)** Movement fractions of 40 movement types in 3D behavioral analysis in the saline group and arecoline group. **(E)** Low-dimensional representation of the two animal groups (saline group, *n* = 10; arecoline group, *n* = 10). The 20 dots in 3D space were dimensionally reduced from 40-dimensional movement fractions, and they are well separated. **(F)** Movement fractions of kinematic parameters comparison in 3D behavioral analysis in the saline group and arecoline group.

The conditioned place preference (CPP) paradigm (Figure 1A) was employed to evaluate the addictive potential of arecoline. Animals were exposed to varying doses of arecoline (0.002 mg/kg, 0.1 mg/kg, and 0.2 mg/kg), and preference changes were assessed pre- and post-conditioning. These doses were selected based on previous studies examining the pharmacokinetic properties and behavioral effects of arecoline in rodents. Notably, prior research has demonstrated that doses of 0.03 mg/kg and 0.1 mg/kg are sufficient to induce significant CPP in adolescent and adult mice (Pi et al., 2023), respectively. In our study, the 0.002 mg/kg dose was chosen as a subthreshold control to assess the minimal effective concentration, 0.1 mg/kg represents a dose previously associated with psychoactive effects, and 0.2 mg/kg serves as a high-dose condition to examine the upper limit of arecoline-induced behavioral changes. Results revealed significant dose-dependent effects. While the low-dose group (0.002 mg/kg) exhibited no significant difference in pre- and post-conditioning scores (two-way ANOVA, Sidak’s multiple comparisons test, *F*_(3, 60)_ = 1.839, *p* > 0.05), the medium-dose (0.1 mg/kg) (two-way ANOVA, Sidak’s multiple comparisons test, *F*_(3, 60)_ = 5.047, *p* < 0.0001) and high-dose (0.2 mg/kg) groups demonstrated marked increases in preference scores (two-way ANOVA, Sidak’s multiple comparisons test, *F*_(3, 60)_ = 5.153, *p* < 0.0001) (Figure 1B). These results suggest that arecoline at medium and high doses has a pronounced ability to induce place preference, indicating its potential for addiction-like behavior in experimental animals.

To further elucidate the behavioral effects of arecoline, 3D motion analysis was conducted to capture detailed activity patterns in the freely moving animals (Figure 1C). Behavioral ethograms were generated based on 40 identified motion characteristics. The arecoline-treated group (0.1 mg/kg) exhibited significant differences from the saline control group. During the first half of the observation period, frequent alternations in movement patterns were observed, which transitioned into prolonged stereotyped behaviors in the latter half.

Distinct postural behaviors such as stationary sniffing and exploratory sniffing (movement type #7, 9, 24) during movement showed significant differences (unpaired *t*-test, *t*_(18)_ = 5.22, *p* < 0.05) (Figure 1D). Additionally, kinematic analysis revealed specific parameters significantly altered in the arecoline group compared to the saline group. These included reduced back movement activity (unpaired *t*-test, *t*_(18)_ = 2.623, *p* = 0.0173), lower left forepaw activity (unpaired *t*-test, *t*_(18)_ = 2.972, *p* = 0.0082), decreased nasal sniffing activity (unpaired *t*-test, *t*_(18)_ = 6.031, *p* < 0.0001), and slower forepaw and nasal movement velocities (unpaired *t*-test, *t*_(18)_ = 4.012, *p* = 0.0008 and unpaired *t*-test, *t*_(18)_ = 6.031, *p* < 0.0001, respectively) (Figures 1E,F). These results indicate that arecoline induces distinct behavioral signatures, suggestive of altered neural activity and motor control.

### Arecoline-induced neural activation of hippocampus

**Figure 2:**
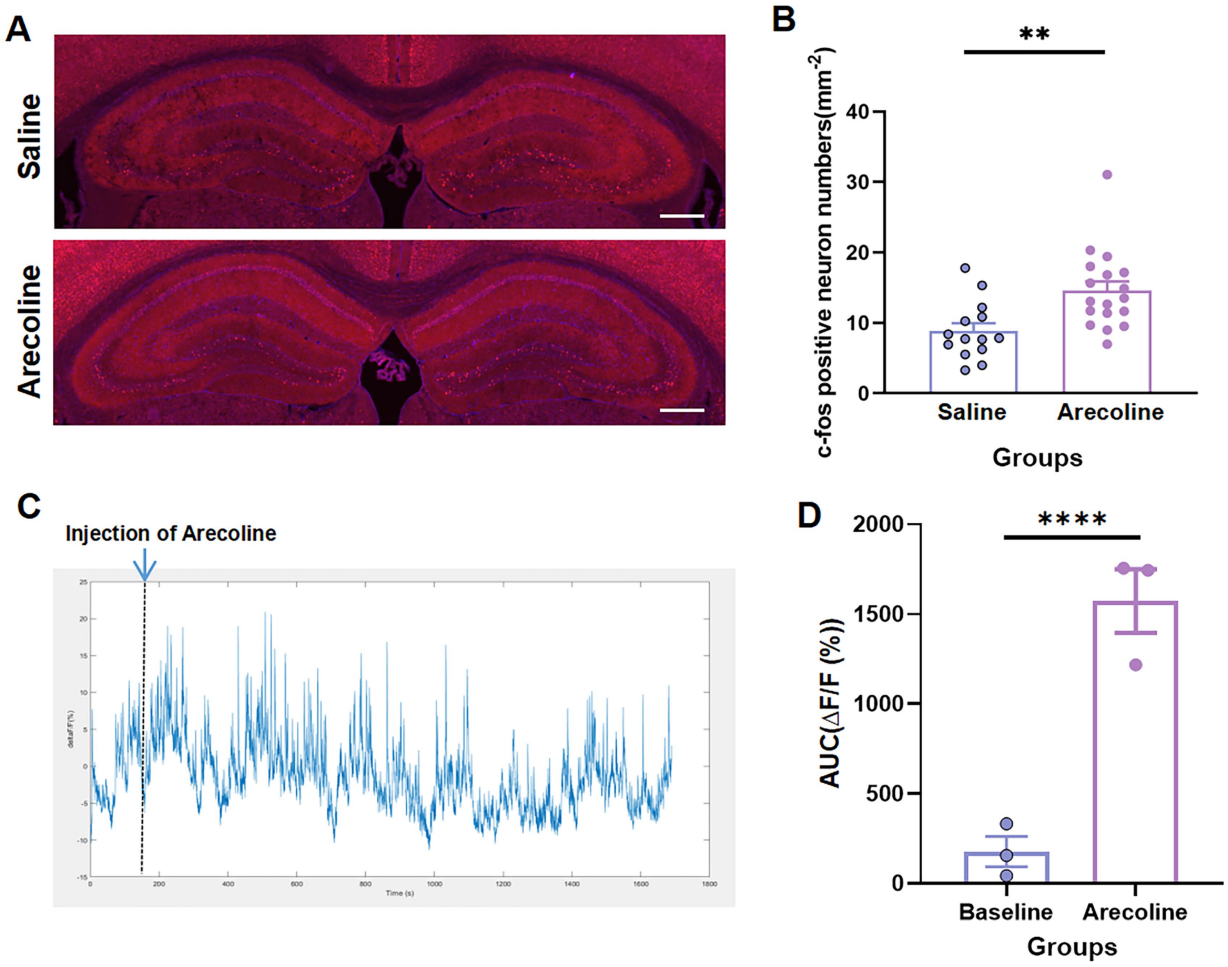
Arecoline-induced neural activation of hippocampus. **(A)** Representative images of c-Fos^+^ neurons of the hippocampus in the saline and arecoline groups. (Top) The c-Fos^+^ neurons of the hippocampus in the saline group. (Bottom) c-Fos^+^ neurons of the hippocampus in the arecoline group. Scale bar: 100 μm. **(B)** The average percentage of c-Fos^+^ in the saline and arecoline groups (unpaired *t*-test, **p < 0.01). **(C)** Representative image of calcium signaling with arecoline effects. **(D)** The average AUC of Δ*F*/*F* calcium signaling with arecoline effects (paired *t*-test, ****p < 0.0001).

No significant changes in activation were detected in the anterior cingulate cortex (ACC); however, the hippocampus exhibited markedly increased neuronal activity, as indicated by a pronounced elevation in c-Fos expression (unpaired *t*-test, *t*_(4)_ = 3.112, *p* < 0.05) (Figures 2A,B). This suggests that arecoline selectively modulates hippocampal circuitry, a region critical for processes such as learning, memory consolidation, and spatial navigation. Dynamic calcium imaging further revealed a sustained increase in calcium signal amplitude within the first 30 min after arecoline administration, followed by a gradual return to baseline levels by 40 min (paired *t*-test, *t*_(2)_ = 11.36, *p* = 0.0077) (Figures 2C,D). This transient yet sustained activation suggests a complex and time-dependent modulation of hippocampal neurons by arecoline. The initial surge in calcium signaling likely reflects enhanced neuronal excitability and synaptic plasticity, potentially linked to the arecoline’s effects on memory formation and cognitive functions. Moreover, the gradual return to baseline activity points to a mechanism of homeostatic regulation, where the hippocampus attempts to stabilize its activity after the transient stimulation. These findings provide important insights into the region-specific modulation of neural circuits by arecoline, with implications for its potential involvement in addictive behaviors and cognitive processes.

### RNA-seq analysis of arecoline-induced changes

**Figure 3:**
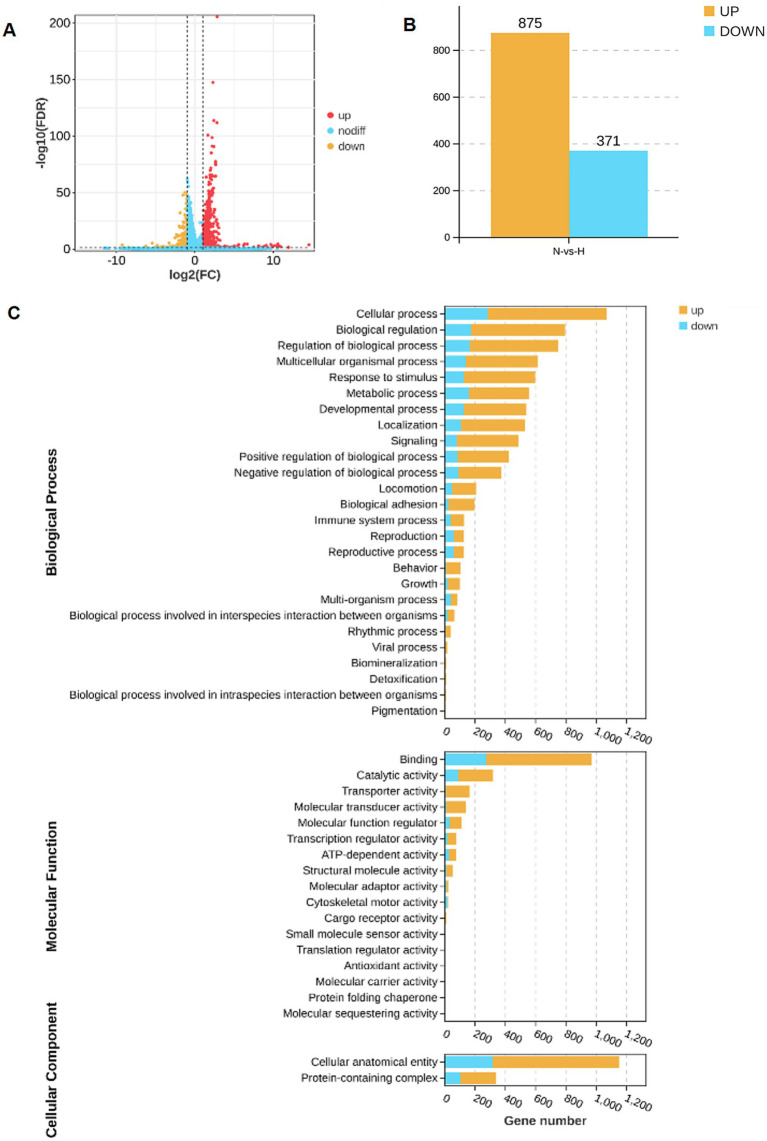
RNA-seq analysis of arecoline-induced changes. **(A)** The volcano plot of upregulated and downregulated genes in the saline-treated and arecoline-treated group. **(B)** The number of upregulated and downregulated genes in the saline-treated and arecoline-treated group. **(C)** The grouped secondary bar chart of Gene Ontology (GO) enrichment analysis.

**Figure 4:**
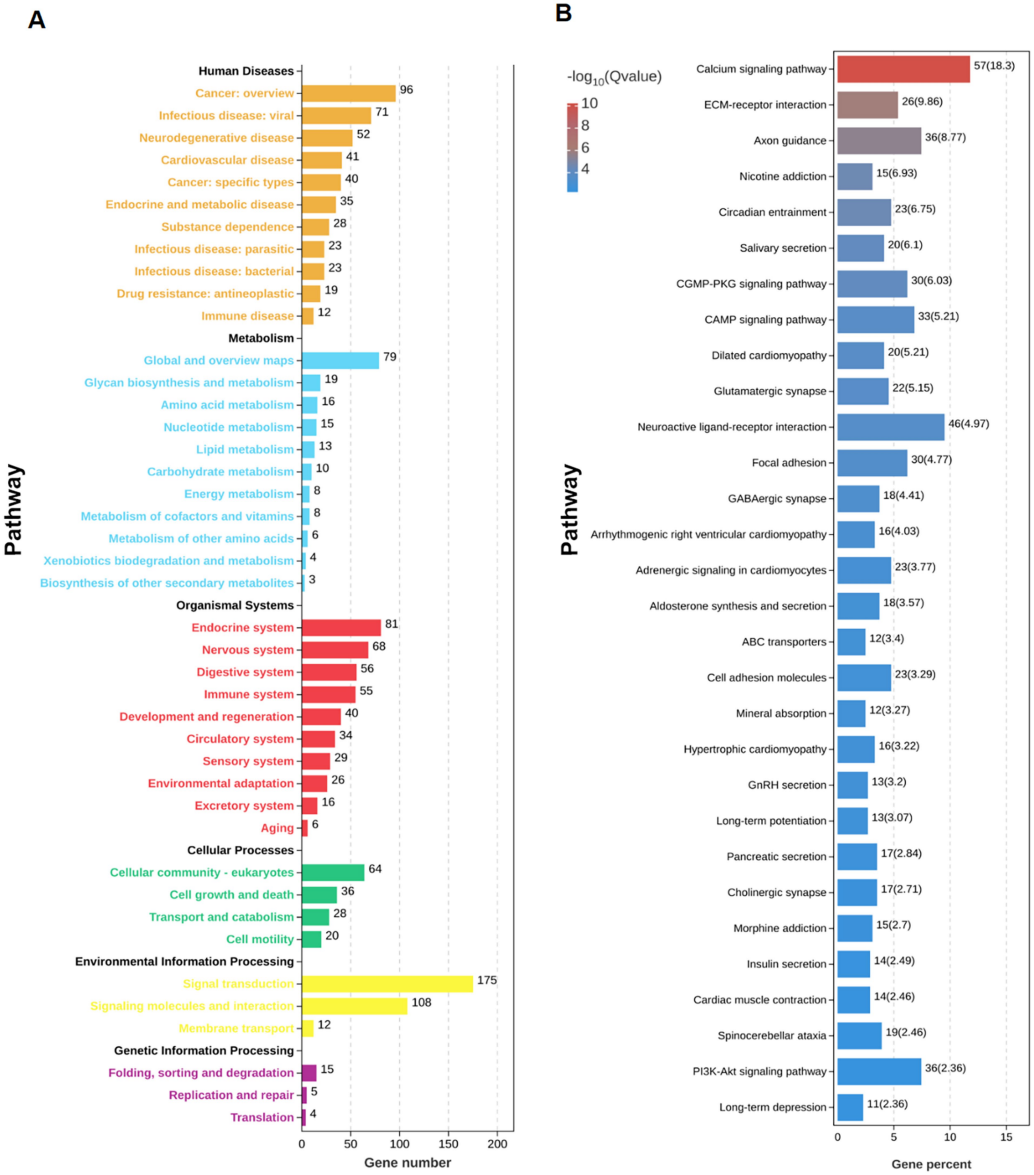
RNA-seq analysis of arecoline-induced changes. **(A)** The number statistics chart of KEGG pathway enrichment analysis. **(B)** KEGG enrichment bar chart between the saline-treated and arecoline-treated group.

RNA sequencing was performed on hippocampus samples from both arecoline-treated and saline-treated animals to investigate changes in gene expression. Differential expression analysis revealed significant alterations in gene expression profiles between the two groups. Volcano plots highlighted a broad spectrum of upregulated and downregulated genes, with several exhibiting high fold changes (Figures 3A,B). Among the top 20 most differentially expressed genes, key alterations were observed in pathways associated with synaptic plasticity, transcriptional regulation, and metal ion transport. Gene Ontology (GO) enrichment analysis identified significant involvement of pathways related to “metal ion transport,” “synaptic structure development,” and “calcium ion signaling,” indicating a potential shift in hippocampal signaling and cellular activity (Figures 3C,D). Similarly, KEGG pathway enrichment analysis highlighted the “calcium signaling pathway” and “synaptic navigation” as being particularly affected (Figures 4A,B), suggesting that arecoline may influence the molecular mechanisms underlying synaptic function and neuronal communication. These findings provide evidence that arecoline induces broad and complex molecular changes in hippocampal gene expression, which are consistent with its behavioral effects and impact on neural circuits, particularly those involved in synaptic plasticity and calcium signaling.

## Discussion

The findings from the current study provide valuable insights into the central actions of arecoline, particularly its effects on the hippocampus (Abbas et al., 2013) and the associated behavioral and molecular mechanisms (Ku et al., 2021). Our results suggest that arecoline exerts a significant influence on neural circuits, affecting both synaptic plasticity and neurotransmitter signaling, and these effects are closely linked to its behavioral impact. The central actions of arecoline, as demonstrated by the behavioral, neural activation, and molecular analyses, offer a deeper understanding of its role in modulating brain function and its potential for inducing addiction-like behaviors.

### Behavioral implications and addiction-like features

Behaviorally, our results from the condition place preference (CPP) paradigm and 3D fine motor analysis suggest that arecoline has addictive potential, as it induced preference and specific behavioral patterns associated with compulsive drug-seeking behavior. The findings from the CPP task indicate that arecoline administration can alter the animals’ preferences for specific environments, which is a hallmark of drug reward and addiction. This behavioral alteration is consistent with its impact on hippocampal activation, as the hippocampus plays a pivotal role in the encoding of contextual and spatial memory related to rewarding experiences (Zeng et al., 2024).

Additionally, the 3D fine motor analysis revealed specific behavioral patterns in arecoline-treated animals, such as altered sniffing behavior and changes in activity levels. These behaviors, including increased sniffing and altered motor movements, suggest that arecoline not only affects cognitive processing but also influences specific motor and exploratory activities that are commonly observed in addiction-like states (Huang et al., 2024). These alterations in movement patterns may reflect the involvement of neural circuits that control both cognitive and motivational aspects of behavior, providing further evidence of arecoline’s addictive potential.

### Neural activation and region-specific effects

One of the key findings of this study is the region-specific activation of the hippocampus following arecoline administration. While no significant changes were observed in the anterior cingulate cortex (ACC), which is often associated with higher cognitive functions such as decision-making and emotional regulation, the hippocampus displayed notable changes in neural activity. This was evidenced by the increased expression of c-Fos, a marker of neuronal activation. The hippocampus is crucial for learning, memory, and spatial navigation, and its heightened activation following arecoline exposure may suggest that arecoline influences cognitive processes related to these functions. The transient increase in calcium signaling observed within the first 30 min after arecoline administration further supports the notion of a dynamic modulation of hippocampal activity, particularly in regions involved in memory encoding and retrieval. The gradual return to baseline levels of calcium signal amplitude by 40 min indicates that arecoline’s effects on neural activation are not persistent but are transient, potentially modulating the brain’s activity in a short-term, yet impactful manner.

These findings are consistent with previous studies showing that cholinergic compounds like arecoline can alter hippocampal function by activating muscarinic and nicotinic acetylcholine receptors (nAChRs), which are heavily involved in synaptic plasticity and memory formation (Sabec et al., 2018). Arecoline’s modulation of the hippocampus suggests its potential role in influencing learning and memory processes (Sabec et al., 2018), which aligns with its known effects on cognitive functions in both animals and humans.

While our study primarily focused on the hippocampus, future research should explore additional brain regions, such as the nucleus accumbens and prefrontal cortex, which are crucial for reward processing and addiction-related behaviors. The absence of data from these regions represents a limitation of the current study, as a more comprehensive understanding of arecoline’s effects on the brain requires whole-brain imaging and region-specific analyses. Future studies employing these techniques will be necessary to fully elucidate the broader neural impact of arecoline beyond the hippocampus.

### Molecular mechanisms and pathway alterations

At the molecular level, RNA sequencing analysis identified significant changes in the expression of genes involved in synaptic plasticity, metal ion transport, and calcium signaling in the hippocampus following arecoline treatment. These molecular changes likely underpin the observed neural and behavioral alterations. The upregulation of genes associated with synaptic structure and plasticity suggests that arecoline may facilitate synaptic changes that contribute to memory and learning processes, which may be dysregulated in addiction.

Notably, the Gene Ontology (GO) enrichment analysis pointed to the involvement of pathways related to “metal ion transport” and “calcium ion signaling.” These pathways are critical for maintaining synaptic function and neuronal excitability, both of which are essential for cognitive and addictive behaviors. The calcium signaling pathway, in particular, plays a crucial role in synaptic transmission and plasticity, processes that are vital for the formation of long-term memory and learning. The involvement of this pathway further emphasizes arecoline’s potential to modulate synaptic activity in ways that could impact both learning and addiction.

Moreover, the KEGG pathway enrichment analysis highlighted “synaptic navigation” as another significantly affected pathway. This suggests that arecoline may influence the guidance and plasticity of synaptic connections, which could explain its effects on behavior and neural activation. Synaptic navigation is essential for proper neural network formation and function, and its dysregulation is often implicated in various neuropsychiatric disorders, including addiction. Therefore, the alterations in this pathway might contribute to the addictive behaviors observed in the arecoline-treated animals.

Additionally, while our study provides evidence that arecoline modulates hippocampal activity, we did not directly assess receptor-specific mechanisms. Future research should further explore how arecoline interacts with muscarinic (mAChRs) and nicotinic acetylcholine receptors (nAChRs) and their implications for synaptic plasticity and neural excitability. These receptors play critical roles in cholinergic signaling, and their involvement in arecoline-induced changes could offer deeper mechanistic insights. We have cited relevant literature supporting these mechanisms and emphasize the need for targeted receptor studies in future investigations.

### Implications for addiction and further research

Furthermore, while our study primarily focused on CPP and 3D motion analysis to evaluate addiction-like behaviors and motor activity, a broader assessment of behavioral effects is necessary. Future studies should incorporate additional behavioral assays, such as the elevated plus maze (EPM) to assess anxiety-like behaviors and novel object recognition (NOR) to evaluate cognitive effects. These additional tests will help provide a more comprehensive understanding of how arecoline influences not only addiction-related processes but also broader neurobehavioral functions.

The changes in gene expression related to synaptic plasticity, calcium signaling, and metal ion transport support the hypothesis that arecoline may have a modulatory effect on neural circuits involved in addiction. The hippocampus, with its critical role in memory and learning, appears to be a central player in arecoline’s effects on both behavior and molecular mechanisms. The combination of behavioral alterations, neural activation, and gene expression changes suggests that arecoline could promote the development of addiction-like behaviors through its influence on hippocampal activity and synaptic plasticity.

Future research should aim to explore the specific molecular targets of arecoline, such as the muscarinic and nicotinic acetylcholine receptors, and their role in the modulation of neural circuits. Additionally, further investigation into the long-term effects of arecoline on synaptic plasticity and its potential to induce persistent changes in brain function could provide valuable insights into its addictive properties. Moreover, exploring the effects of arecoline in other brain regions involved in reward processing, such as the nucleus accumbens and prefrontal cortex, would help elucidate the broader neural mechanisms underlying its behavioral effects.

Moreover, while rodent models provide critical insights into the neurobiological effects of arecoline, translating these findings to human arecoline consumption remains a challenge. Future studies should bridge this gap by considering human-relevant dosages, behavioral parallels, and epidemiological data related to betel quid chewing. The implications of arecoline use in humans, particularly in regions where betel quid consumption is prevalent, highlight the need for translational research to understand its cognitive and addiction-related consequences. This will help inform public health strategies aimed at mitigating the potential risks associated with arecoline exposure.

## Conclusion

In conclusion, this study provides compelling evidence that arecoline induces region-specific neural activation, particularly in the hippocampus, and alters gene expression in pathways critical for synaptic plasticity and calcium signaling. These molecular changes are consistent with the observed behavioral alterations and suggest that arecoline has the potential to influence learning, memory, and addiction-related processes. The combination of neural, behavioral, and molecular data highlights the complex mechanisms by which arecoline may exert its central effects, and offers a foundation for further studies into its potential as an addictive substance.

## Data Availability

The original contributions presented in the study are included in the article/supplementary material, further inquiries can be directed to the corresponding author.
